# Evaluating Community Engagement Supporting LGBTQ+ Health in Schools: Adaptation and Use of the Collaborating with Community Subscale from the Measure of School, Family, and Community Partnerships

**DOI:** 10.21203/rs.3.rs-6735563/v1

**Published:** 2025-06-12

**Authors:** Rachel A Sebastian, Daniel Shattuck, Mary M Ramos, Cathleen E Willging

**Affiliations:** Pacific Institute for Research and Evaluation; Pacific Institute for Research and Evaluation; Pacific Institute for Research and Evaluation; Pacific Institute for Research and Evaluation

**Keywords:** LGBTQ+, community, collaboration, bridging factor, measurement, schools, school health

## Abstract

**Background:**

LGBTQ + youth are at elevated risk for numerous negative health and behavioral health outcomes, which largely stem from minority stress and maladaptive coping. Schools are an important environment where these youth may be exposed to both stressors, like experiences of stigma, bias, discrimination, and violence, and health promotive factors that moderate the impact of minority stress. Collaboration between schools and the broader community plays a crucial role in initiatives designed to improve school climate and culture. In the context of a cluster randomized controlled trial implementing a suite of six LGBTQ + supportive practices in high schools, we used the “Collaborating with Community Scale” adapted specifically to address linkages between schools and communities focused on the needs of LGBTQ + students (CCS-LGTBQ+).

**Methods:**

We conducted annual surveys over five years with an administrator and an implementation leader in each of the 42 high schools randomly assigned to either an implementation condition or a delayed implementation condition. The survey included questions on organizational leadership, implementation climate, and the CCS-LGBTQ+. We analyzed inter-rater reliability between respondent types, internal consistency, and change over time in scale items and means.

**Results:**

Scale scores between administrators and implementation leaders were strongly correlated. However, administrators rated items higher than implementation leaders, as was expected given their relative positions within school communities. The scale demonstrated a high level of internal consistency, with Cronbach’s alphas ranging from .777 to .930 and was sensitive to changes in the implementation of scale items, indicated by increases in the scale means of implementation condition schools from 1.59 in year 1 to 2.08 in year 4 (p < .035).

**Conclusions:**

Testing of the CCS-LGBTQ + resulted in a scale with high internal consistency to measure the extent to which schools collaborate with community resources to support and enhance school environments for LGBTQ + students. When used in the context of the parent trial, findings from the CCS-LGBTQ + show that schools’ collaboration with their communities increased over time. However, the impact of the COVID-19 pandemic likely reversed some of the gains made within the first years of implementation. The CCS-LGBTQ + is a reliable and useful tool for assessing school-community collaboration for supporting LGBTQ + populations.

## Background

Youth who identify as lesbian, gay, bisexual, transgender, queer or questioning, or with other diverse sexual orientations and gender identities (LGBTQ+) experience significant health and educational disparities compared to their heterosexual and cisgender peers. These youth report higher levels of psychological distress, including depression, anxiety, and post-traumatic stress disorder, along with higher rates of self-harm and suicidality (Choukas-Bradley & Thoma, 2022; di Giacomo et al., 2018; Hatchel et al., 2021). They are also more likely to engage in many high-risk behaviors, including substance use and unsafe sexual behaviors (1, 2). Compared to their heterosexual and cisgender peers, LGBTQ + youth face disproportionately high rates of violence and victimization, including physical and sexual assault, physical and sexual dating violence, and bullying (3, 4). These disparities largely stem from minority stress, referring to the compounded effects of discrimination, stigma, harassment, victimization, and lack of belonging, inclusion, support, and affirmation that individuals with marginalized identities experience, often across their lifespan and in various contexts such as family, school, work, and community (5–9).

Research has consistently shown that the mental and behavioral health challenges experienced by LGBTQ + youth are mediated, at least in part, by experiences of victimization and discrimination, as well as protective factors, like family, social, and school support and connectedness (10, 11). Across numerous studies, findings indicate that an inclusive and affirming school environment, supportive relationships with teachers and other adults at school, and involvement in supportive school groups and activities are associated with positive outcomes for LGBTQ + youth (12–18). A growing body of research has demonstrated the potential impact of school climate improvement initiatives on student well-being and academic outcomes (19–22).

Collaboration between schools and the broader community plays a crucial role in initiatives designed to improve school climate and culture (23). Within implementation science, this collaboration can be conceptualized as a bridging factor. Drawing on the Exploration, Preparation, Implementation, Sustainment (EPIS) Framework, bridging factors are conceptualized as factors that connect inner and outer contexts of implementation (24–26). The inner context refers to the specific implementation context (e.g., schools). In contrast, the outer context is conceptualized as the encompassing context or systems in which the inner context is embedded (e.g., public school system, community). Bridging factors are typically characterized as contracts, funding, and memoranda of understanding that can facilitate implementation and support sustainment of a new program, policy, or practice (26). In practice, because community is such a crucial element of successful interventions, it has been built into the foundations of most major school health frameworks like the Whole School, Whole Child, Whole Community model (27).

Methods for assessing community-school collaboration have been developed, but none specifically measure the extent to which community-school collaborations support the needs of LGBTQ + youth (28, 29). As part of a larger project aimed at the implementation of LGBTQ + supportive practices in high schools (30), we developed the LGBTQ + focused Collaborating with Community Scale (CCS-LGBTQ+) to measure school practices for partnering with and integrating resources and services from the broader community to strengthen school programs, learning, and well-being for LGBTQ + students. This tool supports the need for more rigorous measurement and monitoring of key bridging factors for school-based implementation efforts (24).

## Methods

### Parent Study

The CCS-LGBTQ + was developed and used in the context of a five-year cluster randomized controlled trial assessing the process and impact of utilizing the Dynamic Adaptation Process (DAP) for implementing and scaling up six LGBTQ + supportive practices in high schools (30). The DAP is an iterative approach to operationalizing the EPIS Framework that focuses on data-informed and context-specific adaptation and implementation of practices by a local implementation resource team (31). For this study, the DAP supported schools in integrating practices like safe spaces (e.g., Genders and Sexualities Alliances), LGBTQ + inclusive prohibitions on bullying and harassment, professional development for school staff, LGBTQ + inclusive health curricula and materials, and facilitation of access to LGBTQ + affirming medical, behavioral health, and social services providers (32). Table 1 lists the LGBTQ + supportive practices and offers examples of how these can be implemented.

Forty-two schools were enrolled in the trial and randomized into an implementation (intervention) or delayed implementation (control) condition. Those in the implementation condition participated in the DAP and received three years of coaching support. At the end of the three years, those in the delayed implementation condition were provided with one year of support. Participating schools ranged in size from fewer than one hundred to a couple thousand students, and were located across urban, suburban, and rural geographic regions.

As part of the study, site-specific implementation leaders and school administrators at each school site were asked to complete an annual survey assessing individual attitudes and beliefs, perceptions of leadership and implementation support at the school, and implementation efforts related to the LGBTQ + supportive practices. The CCS-LGBTQ + was embedded within this survey to measure respondents’ perceptions of their schools’ connections to and leveraging of community resources in support of LGBTQ + students. The rationale for using the CCS-LGBTQ + was that the types of collaborations it measures would be both a necessary precondition for and a result of implementing the focal LGBTQ + supportive practices within the parent study.

### Adaptation of the Collaborating with Community Scale

The Collaborating with Community Scale was originally developed as a module within the Measure of School, Family, and Community Partnerships, which also includes sections on parenting, communicating, volunteering, learning at home, and decision-making (28). The overall measure is designed as a tool for schools to assess whether they engage parents, community members, and students meaningfully. Its developers recommend that it be used annually to evaluate the progression of any new efforts to engage those groups. The Collaborating with Community Scale elicits information about how schools rate their provision of resource directories for community services, their involvement of families in locating and using community resources, working with local organizations to enhance student learning, offering afterschool programs in collaboration with local organizations, solving “turf problems” within collaborations, and providing a “one-stop shop” for multiple types of family services.

The study team tailored and added these items to reflect an LGBTQ + focus. Recommended changes were shared with members of the study’s Community Academic Partnership (CAP), consisting of local, state, and national school, adolescent, and LGBTQ + health experts. The CAP offered further refinement and simplified language. Through group consensus, the CAP arrived at a final adaptation for piloting. For example, items like “provides a resource directory for parents and students on community agencies, services, and programs” became “provides a directory with information on LGBTQ + community resources.” The item regarding collaboration with community resources to enhance student skills and learning was divided into two items focused on working with local LGBTQ + services and using community resources to enhance the school environment for LGBTQ + students. One item regarding opening school buildings for community use was added to the scale. The total number of items increased from six in the original to eight in the adaptation. Finally, we piloted the adapted subscale with four school sites to check for clarity and applicability of the items. Respondents included school nurses, teachers, counselors, and administrators; their feedback was used to guide additional revisions before including the scale in the school personnel survey. Table 2 shows the original and adapted versions of the scale. In the survey, respondents are asked to select how often each item happens at their school, from one (never) to five (frequently).

### Data Collection

The CCS-LGBTQ was administered as part of an online survey distributed annually between 2017 and 2021 during the spring semester of each academic year. The designated implementation leader and one administrator from each school were asked to complete the survey. Implementation leaders included school nurses, teachers, counselors, social workers, librarians, and other school-based professionals who facilitated implementation resource teams consisting of other school staff to guide the implementation of LGBTQ + supportive practices (see Table 1). Administrators included principals, assistant principals, school directors, or other similar roles. The survey was completed by the same two individuals at each school for each year of the study to the extent possible. In many cases, turnover among school personnel necessitated expanding the sample. The overall survey took 45–60 minutes to complete, and participants received a $50 incentive.

### Analysis

We used SPSS 27.0 to analyze the data. Because respondents were surveyed repeatedly across the five years of the project, only the first record for any given respondent was used when analyzing their demographic and role characteristics. We analyzed inter-rater reliability between respondent types (implementation leaders and administrators) by calculating intraclass correlation coefficients for responses in all years in which a school had one implementation leader and one administrator complete the scale. Internal consistency was measured by calculating Cronbach’s alphas for implementation leaders and administrators separately and combined for all scale items. To assess potential differences between scores at implementation and delayed implementation schools, we averaged ratings across respondents from the same school for each data collection year to create school-level measures.

School-level measures were used to assess change over time within both implementation and delayed implementation schools. Due to the potential impact of the COVID-19 pandemic, we analyzed change over time by looking at differences between years 1 and 4 and years 1 and 5. Year 4 of the project included the academic year 2019–2020. In the spring of that academic year, schools in the project were closed to in-person learning in accordance with public health safety measures after many participants had completed their surveys. Year 5 of the project spanned academic year 2020–2021, during which some schools reopened but had not fully returned to normal operations (i.e., some schools utilized virtual learning, others used hybrid learning models with simultaneous in-person and virtual classrooms, and some that had opened to in-person learning reverted back to virtual learning after reporting COVID-19 cases). Finally, we looked at changes in item scores at implementation schools across years 1 through 5, testing for statistically significant differences between years 1 and 5 and years 1 and 4 for each item of the scale.

## Results

Across the five project years, a total of 368 surveys were collected from 156 unique respondents at 42 schools (Table 3). Of the 42 schools, 22 were implementation schools in which implementation teams were charged with facilitating the implementation of LGBTQ + supportive practices; the remaining 20 schools were delayed implementation schools that received support to implement the practices in year five only. Survey response rates varied by year, and the implementation leaders and school administrators changed at many schools during the project. Additionally, from the original 42 schools, four chose to withdraw from the project: one implementation school in the second year, two implementation schools in the third year, and one delayed implementation condition school in the fourth year. These withdrawals were primarily due to administrative turnover.

The demographic and role characteristics of survey respondents are presented in Table 4. Respondents averaged 47 years of age, most identified as female (68.8%), a minority (13.5%) identified as lesbian, gay, bisexual, or questioning, and 45.8% Hispanic and non-Hispanic white. The majority held a graduate or professional degree (70.1%). Over half of the respondents were the designated implementation leader at the school; the remainder were in an administrator role.

### Community Collaboration Scale

Table 5 shows the item and scale means across all years of the study separately for implementation leaders and administrators and the mean scores for implementation leaders and administrators combined. Cronbach’s alphas were high across all study years for both groups and the groups combined, indicating high internal consistency among the scale items and supporting the use of the items collectively to measure the underlying construct of collaboration between the schools and their surrounding communities.

### Inter-rater Reliability

Overall, administrators tended to score items slightly higher than implementation leaders. This is reflected in low agreement rates between respondent types in Table 6. However, although implementation leaders and administrators had low levels of absolute agreement in their scores, ratings between the two respondent types were correlated, which was reflected in the intraclass correlation coefficients (Table 6). Taken together, this finding suggests that implementation leaders and administrators rated CCS-LGBTQ + items similarly but that administrators generally rated the items slightly higher (as occurring with more frequency) than implementation leaders.

### Changes in Community Engagement

Based on the high alphas across items for each respondent group and overall, supporting their use in construction of a scale of collaboration level, in addition to the intraclass correlation coefficients, reflecting correlated scores between the two respondent types, we averaged scores across respondent types within each school based on the assumption that the “true score” for each item would lie somewhere in between those given by each respondent type. In implementation schools, mean scale scores increased from 1.59 in year 1 to a high of 2.08 in year 4 and then decreased to 1.80 in year 5 (Table 7). The difference in means between years 1 and 4 (1.59 to 2.08) was statistically significant (p = 0.035), but the difference between the year 1 and 5 means (1.59 to 1.80) was not statistically significant, thus implementation schools demonstrated an increase in their overall levels of collaboration with community between years 1 and 4, but this increase diminished in the last year of the project. Changes in scale means across the five years in delayed implementation schools were not statistically significant. Between implementation and delayed implementation schools, mean scores were only significantly different in year 4, at the point in which implementation schools had experienced a statistically significant increase in their CCS-LGBTQ + scores.

Within implementation schools only, mean scores on four specific items of the CCS-LGBTQ + increased significantly between years 1 and 4 (Table 8). Statistically significant increases were reflected in the items regarding providing a directory with information LGBTQ + community resources (year 1 mean = 1.30, year 4 mean = 2.25; p = 0.006); working with students in locating and using LGBTQ community resources (1.55, 2.34; p = 0.014); working with local LGBTQ services, programs, and organizations (1.24, 2.19; p = 0.028); and using community resources to enhance the school environment for LGBTQ students (1.35, 2.12; p = 0.048). However, decreases in the mean scores of these items in the project’s final year resulted in no statistically significant differences in three of the four items in year 5. Only changes in the mean score of the item regarding working with local LGBTQ services, programs, and organizations remained statistically significant (1.24, 1.99; p = 0.007). In addition, the mean score on the item about the school opening its building for community use after school hours decreased significantly in year 5 (2.39, 1.74; p = 0.031).

## Discussion

### Use of the Adapted Community-School Collaboration Scale

Adaptations to the Community-School Collaboration Scale resulted in a scale with high internal consistency to measure the extent to which schools collaborate with community resources to support and enhance the school environment for LGBTQ + students. Utilizing the CCS-LGBTQ + annually with implementation leaders and administrators yielded scores from the two respondent types that were correlated, yet slightly higher among administrators. Interrater discrepancies based on their position or role are not an uncommon occurrence within school-based research (33–35). In a study of perceptions of schools’ emphasis on academic success, researchers found that teachers and principals provided different ratings for the same measures, likely depending on their relationships to the element being measured (36). For example, while they agreed on the school’s overall emphasis on academic success, teachers rated the students’ emphasis on academic success at their schools lower than principals did (36). Given the relative similarity in rating and their approximate relational distance to collaboration with the community between implementation leaders and administrators, the slight differences between their ratings do not invalidate the internal consistency of the tool, but rather underscore the need to pay close attention to who is selected as a rater for specific phenomena (33, 37).

Averaging scores across respondent types produced school-level scores that were useful for assessing the extent to which schools collaborated with community organizations and incorporated community resources in ways that supported LGBTQ + students. Overall, the CCS-LGBTQ + could be a useful tool in implementing projects and programs designed to address school climate and environment through collaborations with community resources to support LGBTQ + student populations.

### Changes in Implementation Schools

Using the adapted CSC-LGBTQ + scale, we measured statistically significant changes within implementation schools over the course of the project, with the results indicating that these indicators of collaboration were increasing until year 4 when the first COVID-19 shutdowns went into effect. Until this point, results indicate a significant increase in community collaboration among implementation schools compared to control condition schools. It is unclear if community collaboration is a necessary precondition or a result of implementing LGBTQ + supportive practices. However, it is clear they are linked. Between years 1 and 4, implementation condition schools increased on the items that were the most aligned with the focal LGBTQ + supportive practices of the study (see Tables 1 and 8). For example, the two CCS-LGBTQ + items focused on a resource directory and working with students to locate and use LGBTQ + community resources aligned with Practices 5 and 6, which together focused on facilitating access to providers not on school property who are experienced in providing social, behavioral health, and sexual and reproductive health services to LGBTQ + youth. In this way, the CCS-LGBTQ + items are reflective of best practices; at the same time, they may function as important implementation strategies for other practices, such as providing safe spaces or professional development for staff. The CCS-LGBTQ + items that had no increase would require larger shifts in how the schools function in their communities and were potentially less directly tied to the LGBTQ + supportive practices. For example, there was no change in the items regarding the schools providing one-stop shopping for LGBTQ+-friendly services or in schools solving turf problems. The items were possibly not as overlapping with the LGBTQ + supportive practices as those above or as necessary for ushering in the practices.

In year 5, changes to school environments to allow return to in-person learning while mitigating virus transmission, alongside changes in the operating of community organizations (continued closures, fewer in-person programs and activities, etc.), resulted in less school-community collaboration. Specifically, the scale item regarding the school opening its building during after school hours showed a statistically significant decline in year 5. This is not surprising given that many school activities and programs remained canceled or modified in light of the continuing COVID-19 pandemic during the 2020–21 school year, and schools would have been unlikely to promote opportunities for in-person gatherings on their property during this time. It is promising that even with the decreases seen in year 5, in particular for items that had shown significant increases through year 4, the item regarding schools working with local LGBTQ services, programs, and organizations remained statistically significantly higher than in the first year of the project. This suggests that despite reducing in-person activities and opportunities, schools continued collaborating with community resources to find ways to support their LGBTQ + students.

### Community Partnership and Implementation

Community engagement and collaboration are overwhelmingly supported as key to the success of schools in general as well as specialized interventions; from the academic success of students to health programs to the implementation of community gardens to overall school improvement, the involvement of community and community partners bolsters efforts (38–47). Within the implementation science literature, community engagement can be conceptualized as a central aspect to the co-creation of interventions and implementation efforts (48), a bridging factor that can positively or negatively influence implementation (49), an implementation strategy used to overcome or leverage a determinant (50), or a combination of the above (51).

Community involvement is such a critical aspect of successful public schools that it is integrated into the foundations of frameworks, such as the Whole School, Whole Child, Whole Community Model that is used throughout the United States to guide the implementation and coordination of school health services (27). Collaboration with the community is also understood to be a universally positive influence for schools when present and a negative influence when absent. The CCS-LGBTQ + measures the presence of supportive collaborations. However, when considering interventions aimed at addressing the needs of stigmatized or marginalized populations, the presence of collaboration can become a mechanism for negative community influence. For example, pressures exerted by socially conservative community environments and the often resigned response of school leadership can constrain the ability of schools to implement LGBTQ + supportive practices (49, 52). The CCS-LGBTQ + then measures characteristics of a supportive version of community collaboration, for which implementation strategies may need to be devised to bolster.

The CCS-LGBTQ + heeds recommendations in the implementation science field to more rigorously measure and describe both the form and function of bridging factors to inform our understanding of their impact on implementation efforts (24). Community engagement in school-based interventions is typically assessed qualitatively and as part of process evaluations (38). Tools like the CCS-LGBTQ + offer a structured way of measuring community collaboration and facilitate comparison across studies and sites, providing a quantitative measure to pair with other data in examining the relationship between collaboration, implementation, and intervention effectiveness outcomes.

While the CCS-LGBTQ + is a potentially reliable measure of the extent to which collaborations are happening, it does not assess why changes occur, whether these types of collaborations were possible in the first place, or what is being achieved through collaborations, all of which are areas for future analysis using this scale. For example, the COVID-19 pandemic’s influence on driving down scores between years 4 and 5 indicates the need to complement the CCS-LGBTQ + with other sources to more fully understand changes in scores. Further, collaborations between schools and LGBTQ + community resources are only possible when there is support from school leadership, appropriate staffing, and available resources in the community with which to engage. This type of engagement may be more difficult for smaller, rural schools than larger, urban schools. These complications underline the utility of the tool while at the same time illuminating the need for integration with other data sources.

### Limitations

There are several limitations to this study. First, the sample size was small and might not be representative of schools in other locations. In addition, staff turnover at the schools resulted in the replacement of implementation leaders and administrators at several schools over the five-year project period. In year 4, data collection was hindered by the COVID-19 pandemic closures and changes in school and community organization operations likely influenced the implementation of LGBTQ + supportive practices and measurement of collaboration between schools and community organizations. Relatedly, community collaboration for the CCS-LGBTQ + was only measured from the perspective of school staff and not community members. Finally, a lack of sufficiently similar comparator scales in the survey preclude examining associations between CCS-LGBTQ + and other scales as an additional measure of validity, which is a point for future research.

### Conclusions

The findings from this study point to the CCS-LGBTQ + as a valuable tool for measuring the extent of school-community collaboration in support of LGBTQ + students. The scale demonstrated high internal consistency and produced meaningful school-level scores that can guide efforts to improve collaboration and resource integration. While minor discrepancies between implementation leader and administrator responses emerged, they align with broader trends in school-based research and emphasize the importance of selecting appropriate raters for specific assessments. The scale’s ability to track changes over time, particularly its sensitivity to shifts brought on by external factors like the COVID-19 pandemic, underscores its utility in monitoring and supporting school climate initiatives.

Beyond simply measuring collaboration, the CCS-LGBTQ + provides a structured, quantitative approach to evaluating how community partnerships influence the implementation and effectiveness of LGBTQ + supportive practices in schools. While the scale does not capture the underlying reasons for collaboration shifts or the full impact of these partnerships, its integration with other qualitative and quantitative measures could offer deeper insights. Future research should explore how contextual factors, such as school leadership support, staffing capacity, and the availability of LGBTQ+-affirming community resources, influence school-community collaboration. Additionally, understanding the unique challenges schools face in different geographic and socio-political environments will be essential to refining strategies that enhance partnerships and ultimately improve outcomes for LGBTQ + students.

## Figures and Tables

**Table 1:** School-Based LGBTQ + Supportive Practices

LGBTQ + Supportive Practices	Example Elements
Practice 1: Prohibit harassment and bullying based on a student’s perceived or actual sexual orientation or gender expression.	District maintains a non-discrimination policy that is explicitly inclusive of students of diverse sexual orientation and/or gender identity or gender expression. District maintains a policy against bullying and harassment that is explicitly inclusive of students of diverse sexual orientation and/or gender identity or gender expression.
Practice 2: Provide “safe spaces” such as the school health office, counselor’s office, designated classroom, or student organization where LGBTQ + youth can receive support from administrators, teachers, other school staff, or other students.	At least one club or group tailored to LGBTQ + students is present in the school, e.g., Gay Straight Alliance/Gender Sexuality Alliance (GSA) or other groups/clubs. School participates in a Safe Zone Program, or similar program, in which staff display posters, stickers, etc., indicating that they are a trained resource-person and/or that their office or classroom is a safe and welcoming space for LGBTQ + students. Notices about LGBTQ+-focused resources (school-based or community) are posted in multiple locations throughout the school. School staff use gender-inclusive and gender-neutral language as a standard practice during classroom instruction and when interacting with students and other school staff.
Practice 3: Provide health education curriculum or supplemental materials that include HIV, other STD/STI, or pregnancy prevention information that are relevant to LGBTQ + youth (e.g., curricula or materials that use inclusive language or terminology).	The health education curriculum or supplemental materials include HIV information with language that is inclusive of LGBTQ + students. The health promotion materials available at the school health office contain health information inclusive of LGBTQ + students.
Practice 4: Encourage staff members to attend professional development on safe and supportive school environments for all students, regardless of sexual orientation, gender identity, or gender expression.	School or district offers at least one annual training that addresses how school staff can contribute to fostering a safe and supportive environment for LGBTQ + students. Resources are disseminated to all school staff to assist them in responding to the needs of LGBTQ + students (e.g. LGBTQ + specific and/or inclusive helplines, online resources, community groups, etc.).
Practice 5: Facilitate access to providers not on school property who have experience providing social and behavioral health services to LGBTQ + youth.	School has a referral guide (paper-based or electronic) that lists social service and behavioral health providers in the community, including those experienced in working with LGBTQ + youth. School has marketing materials (e.g. posters) for community-based behavioral health services (not school-based health centers) that are welcoming and inclusive of LGBTQ + students.
Practice 6: Facilitate access to providers not on school property who have experience providing sexual and reproductive health services (SRHS) to LGBTQ + youth.	School has a referral guide (paper-based or electronic) resource that lists sexual and reproductive health providers, including those experienced in working with LGBTQ + youth. School has staff who can facilitate access to community-based SRHS.

**Table 2:** Original and Adapted Items from the Collaborating with the Community Scale

Original	Adapted
Our school provides a resource directory for parents and students on community agencies, services, and programs.	Our school provides a resource directory with information on LGBTQ community resources
Involves families in locating and using community resources	School works with students in locating and using LGBTQ community resources
School works with local businesses, industries, libraries, parks, museums, and other organizations on programs to enhance student skills and learning.	School works with local LGBTQ services, programs, and organizations
	School uses community resources to enhance the school environment for LGBTQ students
School offers afterschool programs for students with support from community businesses, agencies, and volunteers.	School offers after-school programs for LGBTQ students with support from community resources
School solves turf problems to clarify responsibilities, funding, staff, and locations for community collaborations to succeed.	School solves turf problems of responsibilities, funds, staff, and location for collaboration activities to occur
School provides “one-stop shop” as a full-service school with family services, counseling, health services, recreation, job training, summer programs, and connections with other agencies.	School provides one-stop shopping for LGBTQ-friendly services through partnerships between school and community resources
Other collaborating with the community activities (please describe).	School opens its building for community use after school hours

**Table 3:** Data Collection by Year

	Year 1	Year 2	Year 3	Year 4	Year 5
	2016–2017	2017–2018	2018–2019	2019–2020	2020–2021
Total Surveys Completed	84	79	78	56	71
Participating Schools	42	41	39	34	37
Implementation Schools (vs. Delayed Implementation Schools)	22	21	19	17	18

**Table 4:** Demographic and Role Characteristics of Survey Respondents

	Percent or Mean	Valid N
**Project Role**		
Implementation Leader	56.8%	155
Administrator	42.9%	
**Position Type at the School**		
Administrative	42.2%	154
Health/Behavioral Health	30.5%	
Other	27.3%	
**Education Level**		
Graduate or Professional Degree	70.1%	154
**Age**		
Average Age	46.5	153
**Gender Identity**		
Female	68.8%	154
Male	30.5%	154
Other*	1.3%	154
**Sexual Orientation**		
LGBQ+	13.5%	148
**Race & Ethnicity**		
Hispanic	45.8%	153
Non-Hispanic White	45.8%	153

* Respondents could select multiple categories for gender identity; due to the small sample size and small number of respondents selecting transgender, genderqueer or gender nonconforming, other, and no response, these responses are presented in aggregate.

**Table 5:** Collaborating with Community Scale-LGBTQ+ (CCS-LGBTQ+) Means and Cronbach’s Alphas by Respondent Type

	Year 1	Year 2	Year 3	Year 4	Year 5
**Implementation Leaders**					
School provides a directory of LGBTQ + community resources	1.48	1.52	1.26	2.00	1.83
School helps students locate LGBTQ + community resources	1.59	1.74	1.81	2.26	2.21
School works with local LGBTQ + organizations	1.30	1.48	1.33	2.21	1.88
School provides one-stop shopping for LGBTQ+- friendly services through partnerships with community resources	1.42	1.65	1.11	2.00	1.88
School opens its building for community use after hours	2.19	2.26	2.04	2.21	1.75
School offers after-school programs for LGBTQ + students with support from community resources	0.67	1.13	1.59	1.47	1.21
School solves turf problems of responsibilities, funds, staff, and location for collaboration activities to occur	1.42	1.65	1.59	1.78	1.79
School uses community resources to enhance the school environment for LGBTQ + students	1.22	1.43	1.48	1.95	1.71
**Valid N**	**27**	**23**	**27**	**18**	**24**
**Scale Mean**	**1.42**	**1.61**	**1.53**	**1.99**	**1.78**
**Cronbach’s Alpha**	**0.896**	**0.918**	**0.858**	**0.891**	**0.836**
**Administrators**					
School provides a directory of LGBTQ + community resources	1.70	1.96	1.65	2.26	1.88
School helps students locate LGBTQ + community resources	1.74	2.17	2.04	2.42	2.00
School works with local LGBTQ + organizations	1.41	1.83	1.81	2.37	1.92
School provides one-stop shopping for LGBTQ+- friendly services through partnerships with community resources	1.56	1.70	1.69	2.16	1.75
School opens its building for community use after hours	2.74	2.30	2.81	2.63	2.13
School offers after-school programs for LGBTQ + students with support from community resources	1.04	0.65	1.38	1.58	1.50
**Implementation Leaders**					
School solves turf problems of responsibilities, funds, staff, and location for collaboration activities to occur	2.00	1.57	2.31	1.95	1.92
School uses community resources to enhance the school environment for LGBTQ + students	1.37	1.87	1.69	2.05	1.83
**Valid N**	**27**	**23**	**26**	**19**	**24**
**Scale Mean**	**1.69**	**1.76**	**1.92**	**2.18**	**1.86**
**Cronbach’s Alpha**	**0.833**	**0.777**	**0.901**	**0.888**	**0.930**
**All Respondents**					
**Valid N**	**41**	**40**	**39**	**33**	**36**
**Mean**	**1.55**	**1.63**	**1.64**	**1.83**	**1.76**
**Cronbach’s Alpha**	**0.905**	**0.894**	**0.897**	**0.842**	**0.910**

**Table 6:** Collaborating with Community Scale Measures of Agreement between Implementation Leaders and Administrators by Year

	CCS 1	CCS 2	CCS 3	CCS 4	CCS 5	CCS 6	CCS 7	CCS 8
**Year 1 Scores:**								
**% Agreement**	30.8%	46.2%	30.8%	40.0%	50.0%	53.8%	16.0%	46.2%
**Difference** ^**1**^	−0.192	−0.154	−0.077	−0.040	−0.640	−0.385	−0.440	−0.115
**ICC** ^2^	.500	.405	.577	.182	.363	.642	.373	.703
**95% CI**	−.116, .776	− .326, .733	.057, 0.810	− .857, .639	− .445, .719	.201, .839	− .422, .724	.337, .867
**Year 2 Scores:**								
**% Agreement**	21.7%	30.4%	21.7%	34.8%	33.3%	30.4%	34.8%	21.7%
**Difference** ^**1**^	−0.435	−0.435	−0.348	−0.044	−0.044	0.478	0.087	−0.435
**ICC** ^2^	− .042	−0.281	−0.125	0.352	0.275	0.466	0.369	0.174
**95% CI**	−1.456, .558	−2.021, .457	−1.652, .523	− .528, .725	− .708, .693	− .260, .773	− .489, .732	− .947, .650
**Year 3 Scores:**								
**% Agreement**	24.0%	48.0%	24.0%	48.0%	50.0%	48.0%	20.0%	48.0%
**Difference** ^**1**^	−0.400	−0.200	−0.400	−0.440	−0.760	−0.200	−0.680	−0.160
**ICC** ^2^	0.389	0.500	0.521	0.408	−0.615	0.777	0.337	0.715
**95% CI**	− .387, .731	− .135, .780	− .087, .789	− .344, .739	−2.664, .288	.495, .902	− .504, .708	.353, .874
**Year 4 Scores:**								
**% Agreement**	31.6%	47.4%	36.8%	36.8%	26.7%	21.1%	5.6%	21.1%
**Year 1 Scores:**								
**Difference** ^**1**^	−0.263	−0.158	−0.158	−0.158	−0.421	−0.105	−0.056	−0.105
**ICC** ^2^	0.438	0.457	0.595	0.637	0.517	−0.015	−0.212	0.584
**95% CI**	− .459, .783	− .410, .791	− .052, .844	.057, .860	− .254, .814	−1.634, .609	−2.240, .547	− .080, .840
**Year 5 Scores:**								
**% Agreement**	45.5%	45.5%	36.4%	18.2%	52.6%	45.5%	18.2%	27.3%
**Difference** ^**1**^	−0.05	0.23	−0.09	0.00	−0.50	−0.32	−0.23	−0.09
**ICC** ^2^	0.552	0.749	0.183	0.462	0.489	0.621	−0.062	0.611
**95% CI**	− .080, .814	.396, .896	.−969, .661	− .296, .777	− .231, .788	.088, .843	−1.588, .559	.062, .838

Difference between implementation leader and administrator means; negative values indicate higher scores among administrators.

2 Intraclass Correlation Coefficient

**Table 7:** Community Engagement Between Implementation and Delayed Implementation Schools Over Time

	Year 1	Year 2	Year 3	Year 4	Year 5	Significance (Years 1–4)	Significance (Years 1–5)
**All Community Engagement Scale Items**							
Implementation Schools	1.59	1.62	1.92	2.08	1.80	**0.035**	0.205
Delayed Implementation Schools	1.65	1.71	1.56	1.58	1.77	0.74	0.739
**Significance**	0.550	0.730	0.172	**0.045**	0.935		

**Table 8:** Community Engagement Scale and Item Scores in Implementation Schools Over Time

	Year 1	Year 2	Year 3	Year 4	Year 5	Significance (Y1 – Y4)	Significance (Y1 – Y5)
**Community Engagement**							
School provides a resource directory with information on LGBTQ community resources	1.30	1.53	1.63	2.25	1.85	**0.006**	0.059
School works with students in locating and using LGBTQ community resources	1.55	1.84	2.03	2.34	2.06	**0.014**	0.066
School works with local LGBTQ services, programs, and organizations	1.24	1.51	1.61	2.19	1.99	**0.028**	**0.007**
School provides one-stop shopping for LGBTQ-friendly services through partnerships between school and community resources	1.42	1.56	1.63	1.99	1.71	0.157	0.245
School opens its building for community use after school hours	2.39	2.06	2.44	2.47	1.74	0.893	**0.031**
School offers after-school programs for LGBTQ students with support from community resources	0.83	0.99	1.48	1.32	1.18	0.337	0.294
School solves turf problems of responsibilities, funds, staff, and location for collaboration activities to occur	1.67	1.66	1.94	1.85	1.59	0.654	0.638
School uses community resources to enhance the school environment for LGBTQ students	1.35	1.58	1.61	2.12	1.91	**0.048**	0.075

## Data Availability

Data are available upon reasonable request to the principal investigators (CW and MR).

## References

[1] Goldbach JT, Tanner-Smith EE, Bagwell M, Dunlap S. Minority stress and substance use in sexual minority adolescents: a meta-analysis. Prev Sci. 2014;15(3):350–63. 23605479 10.1007/s11121-013-0393-7

[2] Rasberry CN. Sexual risk behavior differences among sexual minority high school students—United States, 2015 and 2017. MMWR Morbidity and mortality weekly report. 2018;67. 10.15585/mmwr.mm6736a3 PMC6146951 30212446

[3] Price MN, Green AE, DeChants JP, Davis CK. Physical dating violence vctimization among LGBTQ youth: disclosure and association with mental health. J Interpers Violence. 2023;38(15–16):9059–85. 37032552 10.1177/08862605231162655

[4] Ray TN, Lanni DJ, Parkhill MR, Duong T-V, Pickett SM, Burgess-Proctor AK. Interpersonal violence victimization among youth entering college: a preliminary analysis examining the differences between LGBTQ and non-LGBTQ youth. Violence Gend. 2021;8(2):67–73.

[5] Green AE, Price MN, Dorison SH. Cumulative minority stress and suicide risk among LGBTQ youth. Am J Community Psychol. 2022;69(1–2):157–68. 34534356 10.1002/ajcp.12553

[6] Kelleher C. Minority stress and health: implications for lesbian, gay, bisexual, transgender, and questioning (LGBTQ) young people. Couns Psychol Q. 2009;22(4):373–9.

[7] Hatzenbuehler ML, Pachankis JE. Stigma and minority stress as social determinants of health among lesbian, gay, bisexual, and transgender youth: research evidence and clinical implications. Pediatr Clin. 2016;63(6):985–97. 10.1016/j.pcl.2016.07.003 27865340

[8] Goldbach JT, Gibbs JJ. A developmentally informed adaptation of minority stress for sexual minority adolescents. J Adolesc. 2017;55:36–50. 28033502 10.1016/j.adolescence.2016.12.007 PMC5272836

[9] Meyer IH. Prejudice, social stress, and mental health in lesbian, gay, and bisexual populations: conceptual issues and research evidence. Psychol Bull. 2003;129(5):674–97. 12956539 10.1037/0033-2909.129.5.674 PMC2072932

[10] Garcia Saiz E, Sarda V, Pletta DR, Reisner SL, Katz-Wise SL. Family functioning as a protective factor for sexualrisk behaviors among gender minority adolescents. Arch Sex Behav. 2021;50(7):3023–33. 34586546 10.1007/s10508-021-02079-5 PMC9116415

[11] Mitchell KJ, Banyard V, Goodman KL, Strøm IF, Ybarra ML. Exposure to suicidal behavior and social support among sexual- and gender-minority youth. Pediatrics. 2021;147(4). 10.1542/peds.2020-033134 PMC8015153 33722989

[12] Baams L, Russell ST. Gay-straight alliances, school functioning, and mental health: associations for students of color and LGBTQ students. Youth Soc. 2020;53(2):211–29. 39006512 10.1177/0044118x20951045 PMC11244756

[13] Ancheta AJ, Bruzzese J-M, Hughes TL. The impact of positive school climate on suicidality and mental health among LGBTQ adolescents: a systematic review. J Sch Nurs. 2020;37(2):75–86. 33287652 10.1177/1059840520970847 PMC8142116

[14] Kuhlemeier A, Shattuck D, Willging C, Ramos MM. Associations between nonsuicidal self-injury and school-based health-promotive factors for sexual and gender minority youth and their peers. LGBT Health. 2023;10(8):617–28. 37358568 10.1089/lgbt.2021.0404 PMC10734899

[15] Adams BJ, Turner B, Wang X, Marro R, Miller E, Phillips G, Coulter RWS. Associations between LGBTQ-affirming school climate and intimate partner violence victimization among adolescents. Prev Sci. 2021;22(2):227–36. 33219902 10.1007/s11121-020-01192-6 PMC7959187

[16] Russell ST, Bishop MD, Saba VC, James I, Ioverno S. Promoting school safety for LGBTQ and all students. Policy Insights Behav Brain Sci. 2021;8(2):160–6. 34557581 10.1177/23727322211031938 PMC8454913

[17] Abreu RL, Audette L, Mitchell YL, Simpson I, Ward J, Ackerman L, LGBTQ student experiences in schools from 2009–2019: a systematic review of study characteristics and recommendations for prevention and intervention in school psychology journals. Psychol Sch. 2022;59(1):115–51.

[18] Leung E, Kassel-Gomez G, Sullivan S, Murahara F, Flanagan T. Social support in schools and related outcomes for LGBTQ youth: a scoping review. Discov Educ. 2022;1(1):18. 36407890 10.1007/s44217-022-00016-9 PMC9662773

[19] Daily SM, Mann MJ, Lilly CL, Dyer AM, Smith ML, Kristjansson AL. School climate as an intervention to reduce academic failure and educate the whole child: a longitudinal study. J Sch Health. 2020;90(3):182–93. 31903632 10.1111/josh.12863 PMC7427837

[20] Singla DR, Shinde S, Patton G, Patel V. The mediating effect of school climate on adolescent mental health: findings from a randomized controlled trial of a school-wide intervention. J Adolesc Health. 2021;69(1):90–9. 33127241 10.1016/j.jadohealth.2020.09.030

[21] Wong MD, Dosanjh KK, Jackson NJ, Rünger D, Dudovitz RN. The longitudinal relationship of school climate with adolescent social and emotional health. BMC Public Health. 2021;21(1):207. 33485308 10.1186/s12889-021-10245-6 PMC7825179

[22] Tomé G, Almeida A, Ramiro L, Gaspar T, Gaspar de Matos M. Intervention in schools promoting mental health and well-being: a systematic review. Glob J Community Psychol Pract. 2021;12(1).

[23] Anderson-Butcher D, Bates S, Lawson HA, Childs TM, Iachini AL. The community collaboration model for school improvement: a scoping review. Educ Sci [Internet]. 2022; 12(12).

[24] Lengnick-Hall R, Stadnick NA, Dickson KS, Moullin JC, Aarons GA. Forms and functions of bridging factors: specifying the dynamic links between outer and inner contexts during implementation and sustainment. Implement Sci. 2021;16(1):34. 33794956 10.1186/s13012-021-01099-y PMC8015179

[25] Moullin JC, Dickson KS, Stadnick NA, Rabin B, Aarons GA. Systematic review of the Exploration, Preparation, Implementation, Sustainment (EPIS) framework. Implement Sci. 2019;14(1):1. 30611302 10.1186/s13012-018-0842-6 PMC6321673

[26] Lengnick-Hall R, Willging C, Hurlburt M, Fenwick K, Aarons GA. Contracting as a bridging factor linking outer and inner contexts during EBP implementation and sustainment: a prospective study across multiple U.S. public sector service systems. Implement Sci. 2020;15(1):43. 32527274 10.1186/s13012-020-00999-9 PMC7288508

[27] Lewallen TC, Hunt H, Potts-Datema W, Zaza S, Giles W. The Whole School, Whole Community, Whole Child model: a new approach for improving educational attainment and healthy development for students. J Sch Health. 2015;85(11):729–39. 26440815 10.1111/josh.12310 PMC4606766

[28] Epstein JL, Sanders MG, Sheldon SB, Simon BS, Salinas KC, Jansorn NR, School, family, and community partnerships: your handbook for action. Corwin; 2018.

[29] Oakes J, Germain E, Maier A. Outcomes and indicators for community dchools: a Guide for implementers and evaluators. Learning Policy Institute; 2023.

[30] Willging CE, Green AE, Ramos MM. Implementing school nursing strategies to reduce LGBTQ adolescent suicide: a randomized cluster trial study protocol. Implement Sci. 2016;11(1):145. 27770819 10.1186/s13012-016-0507-2 PMC5075193

[31] Aarons GA, Green AE, Palinkas LA, Self-Brown S, Whitaker DJ, Lutzker JR, Dynamic adaptation process to implement an evidence-based child maltreatment intervention. Implement Sci. 2012;7(1):32. 22512914 10.1186/1748-5908-7-32 PMC3436717

[32] Willging CE, Shattuck D, Ramos MM, Richard BO, Lawyer A, Dickson E, Aarons GA. Leveraging the dynamic adaptation process to address LGBTQ + health equity in New Mexico high schools. Front Health Serv. 2025. 10.3389/frhs.2025.1499508 PMC12170598 40529796

[33] Schwab S, Zurbriggen CLA, Venetz M. Agreement among student, parent and teacher ratings of school inclusion: a multitrait-multimethod analysis. J Sch Psych. 2020;82:1–16. 10.1016/j.jsp.2020.07.003 32988457

[34] Holzberger D, Schiepe-Tiska A. Is the school context associated with instructional quality? the effects of social composition, leadership, teacher collaboration, and school climate. Sch Eff Sch Improv. 2021;32(3):465–85.

[35] Kennedy TD, Russom AG, Kevorkian MM. Teacher and administrator perceptions of bullying in schools. Int J Educ Pol Leadersh. 2012;7(5).

[36] Ye W, Scherer R, Blömeke S. Teachers’ and principals’ perceptions of school emphasis on academic success: measurement invariance, agreement, and relations to student achievement. Large Scale Assess Educ. 2024;12(1):19.

[37] Mitchell MM, Bradshaw CP, Leaf PJ. Student and teacher perceptions of school climate: a multilevel exploration of patterns of discrepancy. J Sch Health. 2010;80(6):271–9. 20573139 10.1111/j.1746-1561.2010.00501.x

[38] McMullen JM, George M, Ingman BC, Pulling Kuhn A, Graham DJ, Carson RL. A systematic review of community engagement outcomes research in school-based health interventions. J Sch Health. 2020;90(12):985–94. 33184891 10.1111/josh.12962 PMC7702099

[39] Benjamins MR, Whitman S. A culturally appropriate school wellness initiative: results of a 2-year pilot intervention in 2 Jewish schools. J Sch Health. 2010;80(8):378–86. 20618620 10.1111/j.1746-1561.2010.00517.x

[40] Hoelscher DM, Springer AE, Ranjit N, Perry CL, Evans AE, Stigler M, Kelder SH. Reductions in child obesity among disadvantaged school children with community involvement: the Travis County CATCH trial. Obes (Silver Spring). 2010;18(Suppl 1):S36–44. 10.1038/oby.2009.430 20107459

[41] Johnson-Shelton D, Moreno-Black G, Evers C, Zwink N. A community-based participatory research approach for preventing childhood obesity: the communities and schools together project. Prog Community Health Partnersh. 2015;9(3):351–61. 26548786 10.1353/cpr.2015.0056 PMC4745647

[42] Wright K, Giger JN, Norris K, Suro Z. Impact of a nurse-directed, coordinated school health program to enhance physical activity behaviors and reduce body mass index among minority children: a parallel-group, randomized control trial. Int J Nurs Stud. 2013;50(6):727–37. 23021318 10.1016/j.ijnurstu.2012.09.004 PMC3654538

[43] Langhout RD, Rappaport J, Simmons D. Integrating community into the classroom: community gardening, community involvement, and project-based learning. Urban Educ. 2002;37(3):323–49.

[44] Tucker S, Lanningham-Foster L, Murphy J, Olsen G, Orth K, Voss J, A school based community partnership for promoting healthy habits for life. J Community Health. 2011;36(3):414–22. 20976532 10.1007/s10900-010-9323-9

[45] Zahnd WE, Smith T, Ryherd SJ, Cleer M, Rogers V, Steward DE. Implementing a nutrition and physical activity curriculum in Head Start through an academic-community partnership. J Sch Health. 2017;87(6):465–73. 28463443 10.1111/josh.12515

[46] Kirby MM, DiPaola MF. Academic optimism and community engagement in urban schools. J Educ Admin. 2011;49(5):542–62.

[47] McAlister S. Why community engagement matters in school turnaround. Voices Urban Educ. 2013;36:35–42.

[48] Pérez Jolles M, Willging CE, Stadnick NA, Crable EL, Lengnick-Hall R, Hawkins J, Aarons GA. Understanding implementation research collaborations from a co-creation lens: recommendations for a path forward. Front Health Serv. 2022;2. 10.3389/frhs.2022.942658 PMC10003830 36908715

[49] Shattuck D, Willging CE, Peterson J, Ramos MM. Outer-context determinants on the implementation of school-based interventions for LGBTQ + adolescents. Implement Res Pract. 2024;5:26334895241249417. 38666140 10.1177/26334895241249417 PMC11044576

[50] Metz A, Jensen T, Farley A, Boaz A, Bartley L, Villodas M. Building trusting relationships to support implementation: a proposed theoretical model. Front Health Serv. 2022;2. 10.3389/frhs.2022.894599 PMC10012819 36925800

[51] Pinto RM, Park S, Miles R, Ong PN. Community engagement in dissemination and implementation models: a narrative review. Implement Res Pract. 2021;2:2633489520985305. 37089998 10.1177/2633489520985305 PMC9978697

[52] Shattuck D, Richard BO, Jaramillo ET, Byrd E, Willging CE. Power and resistance in schools: implementing institutional change to promote health equity for sexual and gender minority youth. Front Health Serv. 2022;2. 10.3389/frhs.2022.920790 PMC9979782 36873606

